# Successful Treatment of Recurrent Gastrointestinal Bleeding Due to Small Intestine Angiodysplasia and Multiple Myeloma with Thalidomide: Two Birds with One Stone

**DOI:** 10.4274/tjh.2018.0074

**Published:** 2018-11-13

**Authors:** Ida Hude, Josip Batinić, Sandra Bašić Kinda, Dražen Pulanić

**Affiliations:** 1 University Hospital Center Zagreb, Department of Internal Medicine, Division of Hematology, Zagreb, Croatia; 2 University of Zagreb Faculty of Medicine, Zagreb, Croatia; 3 Josip Juraj Strossmayer University of Osijek Faculty of Medicine, Osijek, Croatia

**Keywords:** Thalidomide, Angiodysplasia, Recurrent bleeding, Multiple myeloma, Antiangiogenic

## To the Editor,

Gastrointestinal angiodysplasia (GIA) is the most common digestive tract vascular malformation, often causing recurrent gastrointestinal bleeding. Despite association with certain hereditary diseases [1,2,3], most GIAs are acquired, associated with aortic stenosis, hemodialysis, malignancies, or liver cirrhosis or idiopathic, and they appear among the elderly (>60 years) [4]. Advances in endoscopy brought about management improvements, but due to numerous lesions disseminated over the digestive tract, treatment of GIA remains a clinical challenge. Novel studies suggested that the use of thalidomide might be beneficial in these patients due to its antiangiogenic properties [5,6]. Thalidomide and its modern analogues currently represent a backbone treatment of another disease: multiple myeloma (MM) [7]. Here we would like to present a case of successful MM and GIA treatment with thalidomide.

Our male patient, born in 1947 and suffering from arterial hypertension, benign prostate hyperplasia, and chronic obstructive pulmonary disease, was diagnosed with symptomatic iron deficiency anemia in 2012. He underwent an extensive gastroenterological workup, which revealed multiple small intestine GIAs causing recurrent bleeding. Several attempts at endoscopic argon-plasma coagulation in the following years were not able to control the disease and the patient required regular blood transfusions (every 3-4 weeks) and parenteral iron supplementation. The patient was referred to a hematologist in 2016 for further assessment. Bleeding disorders were excluded (Table 1), but advanced immunoglobulin G kappa MM was found (ISS 1, with 20%-25% clonal plasma cells in the bone marrow and multiple osteolytic lesions), with no signs of bone marrow or gastrointestinal amyloidosis. Treatment with cyclophosphamide (500 mg/week), thalidomide (100 mg/day), and dexamethasone (40 mg/week) together with monthly zoledronate was initiated in March 2016. Cyclophosphamide was discontinued after 3 applications due to development of paroxysmal atrial fibrillation, requiring thromboprophylaxis with enoxaparin. Six months after treatment initiation the patient achieved a very good partial remission (vgPR) of MM. Owing to age, comorbidities, and the patient’s preferences, he was considered transplant-ineligible and so thalidomide (100 mg/day) and dexamethasone (20 mg/week) were continued. The patient has had no apparent bleeding since March 2016, he has been transfusion-free since October 2016, and he received the last parenteral iron supplementation in October 2017, so GIA endoscopy was not repeated. MM evaluations revealed continuous vgPR after 22 months of treatment; the patient is asymptomatic, suffers no side effects, and continues with thalidomide maintenance (Table 1).

The efficacy of thalidomide as a first-line treatment in combination regimens and as maintenance therapy for MM is well established [8]. Despite the irrefutable success of some novel therapeutic agents, such as proteasome inhibitors and next-generation immunomodulatory drugs, thalidomide still represents a valid treatment choice, especially in countries with limited healthcare resources. Thalidomide has an emerging role in GIA treatment, with shown efficacy in a small randomized trial [5] and multiple case reports (nicely reviewed by Bauditz [6]). Certain patients, especially those with several susceptible conditions as in the case presented here, seem to achieve utmost clinical benefit and improvement in quality of life. The optimal dosage of thalidomide in GIAs is currently not defined, and the side-effect profile might limit its long-term use for disease control. Nevertheless, its efficacy and side-effect manageability make further research worthwhile.

## Figures and Tables

**Table 1:**
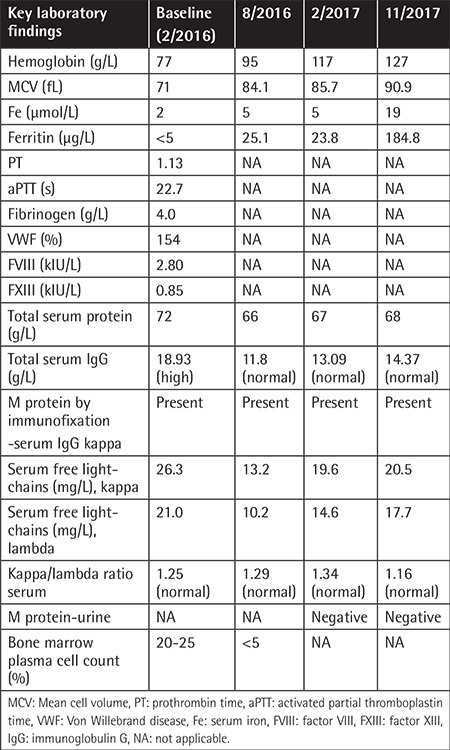
Relevant laboratory findings at baseline and during thalidomide treatment.
